# Understanding the relative risks of zoonosis emergence under contrasting approaches to meeting livestock product demand

**DOI:** 10.1098/rsos.211573

**Published:** 2022-06-22

**Authors:** Harriet Bartlett, Mark A. Holmes, Silviu O. Petrovan, David R. Williams, James L. N. Wood, Andrew Balmford

**Affiliations:** ^1^ Department of Zoology, University of Cambridge, Cambridge, UK; ^2^ Department of Veterinary Medicine, University of Cambridge, Cambridge, UK; ^3^ BioRISC (Biosecurity Research Initiative at St Catharine's), St Catharine's College, Cambridge, UK; ^4^ Sustainability Research Institute, School of Earth and Environment, University of Leeds, Leeds, UK

**Keywords:** agriculture, zoonoses, emergence, livestock, spillover, biodiversity

## Abstract

It has been argued that intensive livestock farming increases the risk of pandemics of zoonotic origin because of long-distance livestock movements, high livestock densities, poor animal health and welfare, low disease resistance and low genetic diversity. However, data on many of these factors are limited, and analyses to date typically ignore how land use affects emerging infectious disease (EID) risks, and how these risks might vary across systems with different yields (production per unit area). Extensive, lower yielding practices typically involve larger livestock populations, poorer biosecurity, more workers and more area under farming, resulting in different, but not necessarily lower, EID risks than higher yielding systems producing the same amount of food. To move this discussion forward, we review the evidence for each of the factors that potentially link livestock production practices to EID risk. We explore how each factor might vary with yield and consider how overall risks might differ across a mix of production systems chosen to reflect in broad terms the current livestock sector at a global level and in hypothetical low- and high-yield systems matched by overall level of production. We identify significant knowledge gaps for all potential risk factors and argue these shortfalls in understanding mean we cannot currently determine whether lower or higher yielding systems would better limit the risk of future pandemics.

## 1. Introduction

Most emerging viral pathogens have zoonotic origins [1]. Zoonotic pathogens are maintained in animal reservoirs and occasionally overcome barriers to spill over and cause pandemics in humans [2]. Recent outbreaks of influenza, SARS, Ebola virus disease and COVID-19 illustrate the devastating impact these can have. The greatest number of zoonotic viruses of concern are found in those domesticated animals which are increasing in abundance (such as pigs and cattle), and in those wild animal species (particularly bats and primates) experiencing displacements and population reductions driven by habitat loss [3].

The risks of emerging infectious diseases (EIDs) are escalating [1], and livestock production plays three key roles in this rise. First, rapidly increasing global demand for animal products means that the total livestock population is now higher than ever and still growing [4]. Livestock biomass now vastly exceeds that of wild mammals and birds, and livestock hosts increasingly outnumber wildlife hosts for pathogens they share [5]. Second, growing demand has in part been met with marked expansion of ‘intensive’ production systems which now provide most of our animal products: 81% of chicken, 61% of pork and 86% of eggs [6]. These systems are reported to be driving EID emergence through risky livestock movements like long-distance live transport, high livestock densities, poor animal health and welfare, low disease resistance and low genetic diversity [7]. Third, escalating animal product demand has also seen the dramatic expansion of land use for livestock and feed production [8]. The resulting conversion and fragmentation of natural habitats means that we are farming in places where livestock and people are intimately associated with demographically disrupted and physiologically stressed populations of wild animals [2,9].

One approach proposed to reduce EID risks is to dramatically reduce meat consumption [10–12]. In the extreme this could allow widespread restoration of natural habitats, increasing the health of wild populations while also greatly reducing opportunities for transmission to livestock and people—hence reducing the risks of disease emergence. However, given long-term trends in per capita wealth and robust relationships between income and consumption of livestock products [13,14], reducing livestock demand substantially is likely to be extremely challenging [15]. This means it is important to determine how any non-zero demand for livestock products can be met at least cost in terms of EID risks. Many argue this could be best achieved by the widespread adoption of less ‘intensive’ production practices [16–20]. While intensification is a poorly defined term, used to refer to many different dimensions of system change, it is commonly linked to increased farm yields (production per unit area). A reduction in production ‘intensity’ is therefore likely to reduce yield, and as a result more land must be farmed to meet a given level of production [21]. This, in turn, could increase those aspects of EID risk associated with the extent, condition and distribution of natural habitats [22].

**Figure 1.**
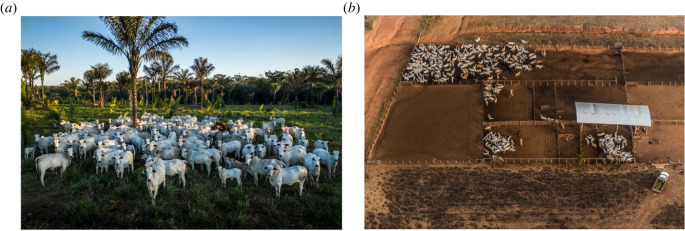
Contrasting beef systems in Brazil. (*a*) low-yielding pasture adjacent to rainforest and (*b*) a high-yielding feedlot system. To understand the relative EID risks of each system, it is important to consider the risks associated with both livestock management and land use. Photos reprinted with permission from Fábio Nascimento.

The importance of production intensity can be illustrated by examining projections of future agricultural land use. A recent study [23] found that a business-as-usual scenario (with no reduction in animal product consumption and slight efficiency gains as projected by Bajželj *et al*. [24]) could require an additional 12.5 million km^2^ of farmed land between 2009 and 2050. This is likely to come at the expense of natural ecosystems [25]. If instead ‘intensive’ livestock systems were used, 31% less land than the business-as-usual scenario would be needed by 2050 [23]. Further illustration comes from regional case studies. For example, Brazilian beef production is expected to rise by approximately 14% between 2000 and 2040 [26]. At current yields this could require approximately 140 000 km^2^ of additional cultivated pasture [26], while if production was switched entirely to less intensive, lower yielding systems, this rises to 570 000 km^2^. However, by increasing pasture productivity to 70% of its carrying capacity, future demand could actually be met on 360 000 km^2^ less land than in 2000, and if pasture yields could match those achieved in recent large-scale trials [27] an even smaller area could be required. Thus, while lower and higher yielding approaches to livestock production clearly differ in many ways that are consequential for the relative risks of EIDs (see the two examples shown in figure 1), the land area they require will have profound consequences for the likelihood of EID emergence. To determine how to minimize the likelihood of future EIDs, we must thus understand the relative importance and sensitivity of both management- and land use-related risks.

Here we assess the state of knowledge on this crucial topic. We identify those attributes or consequences of livestock production systems that influence EID risk, and summarize, for each of these factors, the evidence linking it to EID risk. We also explore what is known about how risks might vary with yield. Importantly, we explicitly consider those factors linked to land use as well as those linked to livestock management. This more holistic framing of the diversity of ways in which farming may influence zoonosis emergence provides a powerful new perspective for evaluating the overall EID risk of different approaches to meeting demand for livestock products.

## 2. Scope and approach

We define EIDs as pathogens whose incidence in human populations has increased within the past two decades or may increase soon [28]. We focus on potential viral zoonotic EIDs that could infect the major terrestrial livestock species groups (cattle, small ruminants, pigs and poultry), or be affected by their production—for example, via livestock-driven habitat loss. We define EID risks in relation to the risk of emergence in livestock and human populations [2]. We consider factors related to the likelihood of spillover, take-off or the potential severity of their consequences. Ideally, we would use a standardized empirical framework to quantify EID risk, but unfortunately there are too many data gaps in the direction and magnitude of EID risks—which vary by system, pathogen and region. Our insights are therefore generalized and not specific to any given system. Due to the size of the literature (e.g. a Google Scholar search on ‘emerging infectious disease’ and ‘agriculture’ returned 271 000 highly diverse papers in February 2022) and the broad focus of this study, it was beyond the scope of this study to undertake a systematic review. Instead, a search of the literature was performed to identify relevant primary and secondary literature, which was then selected subjectively. A list of literature cited in §§4–8 is included in the electronic supplementary material, Appendix S1, with summaries of the relevant conclusions and limitations of each paper. In identifying risk factors to focus on we were informed by our own views, as authors of a recent review and solution scan of options for preventing future zoonotic epidemics, conducted with a large group of multidisciplinary experts [29], and other papers [10,18,19]. We focused on identifying risk factors that relate to fundamental principles in transmission dynamics linked to livestock management practices and land use. We believe that EID risk can only be meaningfully assessed by comparing contrasting livestock systems matched by level of production, or using units of production as a denominator, for example, ‘risk per tonne of meat produced’. Failing to do this makes it impossible to determine which system could realistically meet any given level of demand with least EID risk. For this reason, we do not consider EID consequences arising from changes in the overall level of livestock production, or in the relative proportion of different livestock products, which are also likely to have important consequences for EID risk [22]. We also recognize the importance of bacterial and invertebrate-vector-transmitted zoonoses, and the indirect effects of livestock production on wild meat, nutrition and human immunocompetence [30,31], but for pragmatic reasons do not focus on these here. Climate change will impact livestock systems and associated EID risks in ways that will vary by context and system type. This adds further complexity, but again we do not focus on this here.

To develop a systematic overview of what we know and do not know about the lowest risk approach to meeting livestock demand we need a means of classifying contrasting production systems. Traditional management-based classifications—such as by housing type, by scale, by involvement in labelling schemes or by the terms ‘intensive’ and ‘extensive’—often overlook variation in factors which may be important determinants of EID risk. For example, most characterizations of ‘intensive’ or ‘extensive’ systems do not consider the land used to grow feed, which may influence EID risk through its impacts on extent, condition and distribution of natural habitats. To ensure we include risk factors associated with both management and land use, we classify contrasting systems by their yield—their production per unit area, including land used to produce feed and rear animals. Framing systems by yield has helped shed light on other impacts of livestock and crop farming [32], with evidence from five continents and more than 2500 species revealing that impacts on biodiversity and greenhouse gas fluxes would be best limited by combining high-yield production with off-farm retention or restoration of large tracts of intact habitat [33–39]. Livestock systems are highly contextual, with substantial variation between livestock species, production type (e.g. meat versus dairy) and region. What is classified as ‘high-yield’ production in one sector may be far lower yielding than ‘medium-yield’ production in another. It is therefore important to ensure that comparisons are made between comparable systems, rather than say, Brazilian commercial beef production and Southeast Asian backyard poultry production. In livestock production, yields typically increase from backyard and free-range systems with poor-quality diets, to free-range systems with improved diets, to semi-confined and confined systems. High yields are often associated with ‘factory farms’ but this need not be the case—as illustrated by the similarly high yields of some Brazilian silvopasture and feedlot beef systems [32]. In this review, we attempt to harmonize management and yield-based classifications, albeit roughly, framing our analysis in the context of yields but using examples characterized by management type. When we use examples by management type we place these terms in quotations and estimate the relative yields where possible.

## 3. A heuristic for exploring how risk factors vary

**Figure 2.**
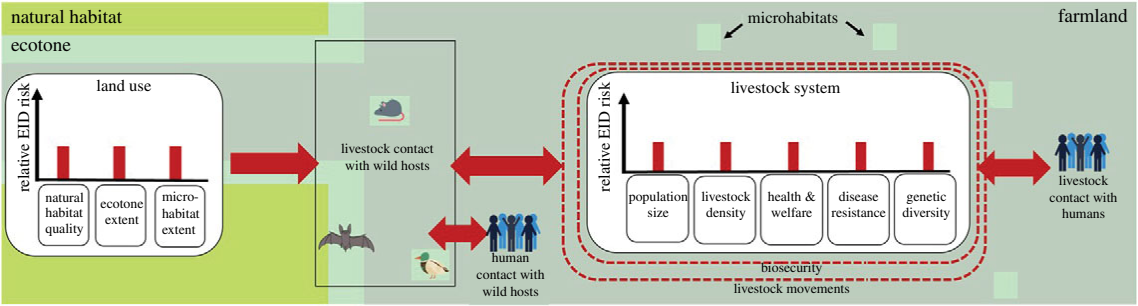
Schematic diagram of the different EID risk factors associated with livestock production for a mix of production systems chosen to broadly reflect the current livestock sector at a global level. Green areas represent natural habitat, grey areas represent farmland and turquoise bands are the ecotones between them. Turquoise squares represent microhabitats. This scenario is used as a baseline, with the size or width of red bars, lines and arrows (describing the approximate magnitude of risk factors and contact rates) set to reflect intermediate levels of risk. ‘Natural habitat quality’, ‘health and welfare’, ‘disease resistance’, ‘genetic diversity’ and ‘biosecurity’ are negatively associated with relative EID risk, so relative EID risks are greater (and red bars, lines and arrows larger) when these attributes are lower (e.g. when natural habitat quality, and health and welfare are poorer). The opposite is the case for all other risk factors—so risks (and red symbols) are greater as, for example, ‘ecotone extent’, ‘human contact with wild hosts' and ‘population size’ increase.

**Figure 3.**
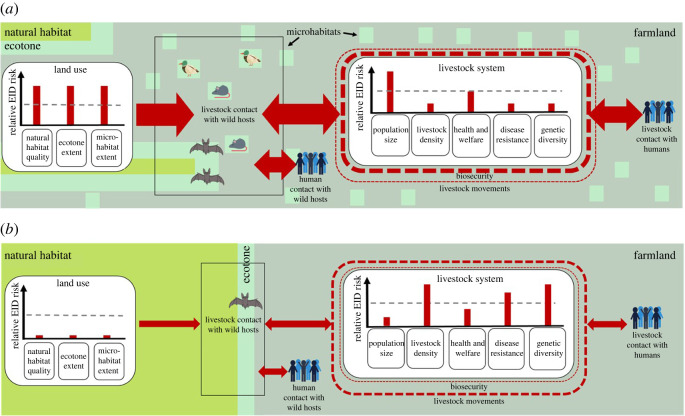
Schematic diagram of how different EID risk factors associated with livestock production might vary under contrasting approaches to meeting the same level of livestock product demand as figure 2, which is taken as a baseline. The dashed grey lines serve as a reminder of the relative EID risks of the figure 2 baseline. These two panels show how EID risks might change if instead the same level of demand was met using low-yield (*a*) or high-yield systems (*b*). Relative EID risks are described by the size or width of red bars, lines and arrows.

The schematic diagram in figure 2 illustrates how key EID risk factors may play out for a mix of systems chosen to broadly reflect the current global livestock sector. The main risk factors directly linked to management practices (references below) are biosecurity, livestock movements, livestock population size, livestock density, livestock health and welfare, disease resistance and genetic diversity. Factors that impact EID risk through land use are the extent, condition and distribution of natural habitats, ecotones (defined here as transition zones between natural habitats and anthropogenic land-covers) and on-farm microhabitats where livestock and wild species that may harbour pathogens interact. We take the scenario in figure 2 as a baseline, with the size or width of red bars, lines and arrows describing the relative magnitude of risk factors and contact rates, set to reflect intermediate levels of risk. The two panels in figure 3 show how EID risks might change if the same level of demand was met using low-yield (figure 3*a*) or high-yield (figure 3*b*) livestock systems, with the relative sizes of the red symbols in these two schematics representing how the scale of each EID risk might compare with the baseline.

In §§4 to 7, we examine each of these EID risk factors in turn. We evaluate the evidence for the link between each risk factor and the risk of EID emergence, and consider how the risk might vary with yield. In §8, we then return to figure 2 to consider, at the level of entire systems, what conclusions might be drawn about how EID risks compare between higher and lower yield systems.

## 4. Biosecurity, livestock movements and their consequences for contacts between livestock, people and wildlife

Biosecurity is defined as the set of measures put in place to prevent the spread of potentially harmful biological substances and contaminants—in this case pathogens with the potential to cause EIDs. Improved biosecurity reduces EID risk by minimizing and managing direct and indirect contacts between livestock, people and wildlife [40–42]. These measures apply to everything that enters and leaves the farm and include practices like disinfection, workers changing clothes and using personal protective equipment, compulsory livestock and worker quarantines and minimizing the size of livestock groups [29]. In addition to the risks of transmission within a farm, livestock movements to and from farms can present opportunities for risky contacts and can play an important role in disease transmission [43]. For example, the spread of the global live pig trade strongly predicts the spatial dissemination of swine influenza A [44].

Studies on biosecurity practices and EID risk typically focus on a few practices or systems and have small sample sizes and restricted geographical range [45,46]. The limited evidence available reveals a complex picture of how risks might vary. In general, ‘extensive’, ‘hobby’ and ‘free-range’ (typically low-yield) systems have poorer farm biosecurity allowing a greater number of direct and indirect contacts with wildlife [10,47,48] compared with indoor or ‘intensive’ systems [46,49–51]. Low-yield (especially ‘backyard’) systems are also more likely to engage in specific high-risk, poor-biosecurity practices, such as the feeding of untreated catering waste [52] and allowing interspecies mixing both on and off farm [53,54]. In addition, low-yield systems have higher labour requirements [55] which requires more dwellings spread across larger agricultural areas and hence higher rates of human–livestock contact. Poultry systems described as both ‘industrial’ and ‘backyard’ played a role in the 2004 outbreak of highly pathogenic avian influenza (HPAI) in Thailand [56,57]. It is debated which played a greater role in the outbreak: spillover in ‘backyard’ production due to poor biosecurity permitting contact between wild and domesticated birds [56], or amplification and reassortment from low to high pathogenicity in ‘industrial’ systems [57].

Large-scale production (which is typically high-yield) is associated with more and longer distance livestock movements during trade and ‘free-range’ or ‘hobby’ systems with shorter movements [12,46,58]. However, this is not consistently the case, as illustrated by the growing regionalization of farms, slaughterhouses and processing plants, which is reducing transport distances in typically high-yield systems [59], and the long-distance transport linked to some low-yield systems. For example, beef from low-yield ranches in the Amazon is reaching European markets [60].

## 5. Livestock population size, livestock density and health and welfare

Livestock population size, density and health and welfare can all influence zoonosis spillover and transmission. A larger livestock population—on individual farms and overall—presents a larger potential host population with more opportunities for contact and transmission within and between farms, which in turn can drive greater pathogen diversification. Population size and livestock density can interact to have a synergistic effect on EID risk, as seen with bovine tuberculosis incidence [61] and swine influenza persistence [62] increasing with herd size in the UK and The Netherlands, respectively. Livestock density has two components: stocking density (the density of livestock within a landscape) and farm density within a landscape—both of which are positively associated with higher EID risks. For example, the risk of HPAI reassortment (and hence take-off in poultry, spillover and take-off in people) was predicted by poultry and human population densities [63], and regions with high densities of pigs had higher seroprevalence of swine influenza [64]. As stocking density increases and transmission is more frequent, the cost of virulence to the pathogen declines [65]: high stocking density thus allows more virulent pathogens to persist and take off [57,66]. However, this is not universally true and evolution of virulence (or the reverse) is a disputed area, as evident from much of the debate over the course of the COVID-19 pandemic. Interestingly, modelling of Marek's disease found that high chicken stocking density selected for less virulent strains [67]. High farm density within a landscape is associated with an increased probability of inter-farm transmission and hence take-off within the livestock population and spillover into people. Poor livestock health and welfare can cause immunosuppression [68] and facilitate pathogen shedding and transmission [69], although the mechanisms underlying this are poorly described.

These risk factors may vary with yield in several ways. Slower growth rates and smaller yields per animal mean that low-yield systems require a larger livestock population to meet a given level of production [70]. ‘Indoor’, ‘intensive’ and ‘industrial’ (typically high-yield) systems involve fewer livestock and farms (for a given level of production) but are more likely to have larger populations per farm, kept at higher stocking densities. Meadows *et al*. [71] found that stocking density could be more important than farm density in driving outbreaks of foot and mouth disease in the US and hence expressed concern about the EID risk of fewer, larger farms. However, it is important to note that this analysis involved holding cattle population size (rather than level of production) constant and so did not account for productivity differences which might have meant that large farms could meet a given level of production with fewer livestock overall. In addition, this trend is not universal: some low-yield systems such as ‘extensive’ ruminant ranches have very large herd sizes, and some high-yield farms keep animals in small groups designed to minimize disease transmission. ‘Intensive’ systems and ‘factory farms’, which are typically high-yield, have been linked with relatively poor livestock health and welfare [72], but the limited evidence for this comes largely from small-scale studies of potentially unrepresentative systems [73] in part due to the challenges of conducting research in commercial farms [74]. Indoor farms are often associated with high stocking densities and relatively poorer health and welfare standards, including increased aggression, stress and injury [75,76], though ‘extensive’ (typically low-yield) systems are associated with different welfare problems [77,78] including increased neonatal mortality, parasitic burdens, thermal stress, predation, hunger and thirst [48,79]. It is important to note that win-win for health and yield are possible via, for example, reductions in mortality and improved animal health [80].

## 6. Disease resistance and genetic diversity

Disease resistance is defined as the ability to limit pathogen burden [81] and transmission. Disease resistance is both innate and adaptive [82] and varies within and between breeds of livestock, for example, zebu are more resistant to tuberculosis than Holstein-Friesian cattle [83] and Haringhata black chickens have better B-cell mediated immunity than other breeds [84]. The susceptibility of all breeds to a novel EID is likely to be high and differences between breeds unpredictable, perhaps with the exception of vector-borne and re-emerging diseases. For the latter, high levels of innate disease resistance or immunity in a farm may confer resistance and potentially impede re-emergence. There is, however, evidence that sometimes the opposite is true—partially immune populations can have longer and larger outbreaks [16]. Livestock genetic diversity could have either positive or negative impacts on EID risk. Pathogens may diversify more when exposed to a greater genetic diversity of host species [85], but it is uncertain the extent to which this also applies to within-species genetic diversity. It is plausible that high within-species diversity might also increase pathogen diversification and hence EID risk. By contrast, livestock genetic diversity can also provide resilience to disease [86,87], but again data supporting this largely relates to endemic diseases.

‘Intensive’ (typically high-yield) livestock production is associated with a narrowing genetic resource base of highly productive breeds [88–90], whereas ‘backyard’ systems often have higher genetic diversity and primitive breeds that are resistant to endemic infectious disease [58,91]. For example, low-yielding N'Dama cattle native to West Africa are resistant to trypanosomiasis whereas other cattle breeds are not [92]. It is important to note that ‘intensive’ systems now increasingly value a broader suite of traits, and there is ongoing work identifying and introducing key resistance genes into high-yielding breeds [92]. Low-yield, especially ‘backyard’, systems generally keep animals on the farm for longer due to slower growth rates and smaller incentives to cull animals when productivity declines [58]. This may result in farmers both choosing and retaining animals that have greater genetic diversity, potentially driving pathogen diversification while also conferring resistance to vector-transmitted or re-emerging EIDs [57]. It is difficult to disentangle how these countervailing risk factors might play out and hence the overall effect of livestock diversity on EID risk.

## 7. Natural habitats, ecotones and on-farm microhabitats and their consequences for contacts between livestock, people and wildlife

Livestock rearing and feed production use 77% of agricultural land [4] equating to 39% of all habitable land on Earth. Livestock production therefore greatly influences three key factors in determining the rates and riskiness of contacts between livestock, people and wildlife: the extent, condition and distribution of natural habitats, the extent of ecotones between natural and managed lands, and on-farm microhabitats

All three factors influence EID risks through similar mechanisms: by altering ecological dynamics [93] to favour generalist species, which are more likely to host zoonoses [94], have higher rates of pathogen shedding [95,96] and increased contact rates between wildlife, humans and livestock. However, studies quantifying causal links between habitat change, physiological stress and pathogen shedding are rare and limited in their spatial distribution and replication [95].

The expansion of land use for livestock production is a major driver of natural habitat loss with, for example, pasture expansion for beef causing 41% of tropical deforestation between 2005 and 2013 [8]. Habitat loss and the accompanying fragmentation also increase the extent and distribution of ecotones, defined here as transition zones between natural habitats and anthropogenic land-covers. Theoretical modelling has shown that ecotone extent is positively correlated with interspecies contact rates and pathogen sharing between wildlife and humans [9]. Ecotones are believed to have had a role in the emergence of yellow fever, Nipah virus disease, influenza [97] and Ebola virus disease [98], but causative evidence is lacking. Conversely, restoring natural habitats may reduce EID risk by improving their extent, condition and distribution and reducing the extent of ecotones. For instance Prist *et al*. [99] report that restoration of the Brazilian Atlantic Forest could reduce the abundance of two reservoir host rodent species and hence the transmission of hantavirus cardiopulmonary syndrome to people by approximately 45%. However, effects are likely to vary with agricultural and ecological system—for instance restoration in some temperate biomes has been correlated with increases in zoonotic disease [100].

Microhabitats (small-scale habitat features) on farms are also implicated in increased disease risk [101,102] by attracting and providing resources for generalist host species, and thereby increasing their contact with livestock and people. Microhabitats and habitat loss have together played a central role in the spillover of Nipah virus into people in Bangladesh and pigs and subsequently people in Malaysia [103,104], and Hendra virus into horses and subsequently people in Australia [97]. In the case of Hendra virus, large-scale deforestation has removed key food resources and shifted *Pteropus* bat roosting and feeding sites into urban and agricultural areas. As a result, nutritionally stressed bats in trees in farmed areas now exhibit increased viral shedding, resulting in a greater transmission risk of Hendra from bats to nearby horses [105].

Higher yield livestock systems, by definition, have smaller land footprints for any given level of production, hypothetically allowing for the greater retention and restoration of natural habitats [25]. By reducing both habitat loss and fragmentation and the extent of ecotones, higher yielding systems thus have the potential to reduce wildlife–livestock–human interactions, and the prevalence of ecological dynamics that promote EIDs. Livestock in such systems are also more likely to be housed or kept in secure enclosures removed from natural habitats, further reducing interactions. Many microhabitats such as ponds and stands of trees are also less likely to be retained in higher yielding systems, again reducing wildlife–livestock interactions. However, these benefits are not guaranteed: often specific market or policy mechanisms will be needed to actively link yield increases with habitat conservation [39,106]. Data are also needed on the relative importance of specific microhabitats for spillover: some microhabitats such as buildings and farm stores may be more prevalent in high-yield operations and may support generalist species thought to be particularly important reservoirs of novel pathogens [94,102].

## 8. Contrasting systems compared

We now return to the scenarios shown in figures 2 and 3 to explore how all of the risks just considered might play out at landscape scale. The scenario in figure 2 is used as our baseline, which shows EID risks from a mix of systems chosen to generally reflect our current global livestock sector. Figure 3 shows the relative risks associated with low- (figure 3*a*) and high- (figure 3*b*) yield systems. Low-yield systems require a larger livestock population and workforce spread across more farms. These farms frequently have relatively poor biosecurity but may typically keep livestock with greater genetic diversity at lower stocking densities. The consequences for disease resistance, health and welfare standards and livestock movements are likely to be heterogeneous and context dependent. Due to lower yields, a greater total area of land is required to produce a given amount of livestock product, reducing space for natural habitats while increasing fragmentation and the extent of ecotones and microhabitats on farms. This, in combination with a greater chance of livestock and farmers entering risky, poorly regulated areas permitting interspecies contact increases the risk of spillover.

The relative EID risks for high-yield systems are strikingly different. Greater biosecurity and reduced exposure to pathogen sources at ecotones and in on-farm microhabitats are all likely to lower risks of spillover, relative to those in lower yielding systems. However, subsequent risks of take-off may be higher due to greater stocking and farm densities, lower genetic diversity of livestock, and at least in some cases poorer health and welfare. The relative merit of each system may therefore hinge on the extent to which low-yield systems can overcome risks associated with spillover, and high-yield systems can overcome risks associated with take-off.

In considering which system might carry least risk, there are two additional considerations. First, it is important to note that an intermediate system, or a mixture of both systems, could present higher risks all-round if poor biosecurity and long-distance live transport is combined with increased contact with wildlife [10]. Second, it is essential to look beyond current systems to identify significant and feasible improvements. Many of the main risk factors for higher yield systems could be more easily overcome [41], for example, through technological innovations (e.g. biosecurity measures, such as the routine isolation of groups within a farm regardless of disease status to reduce transmission opportunities and take-off), aided by the greater capital investment and economies of scale in these systems. By contrast, the key risks associated with lower yield systems, such as poor biosecurity, may be more fundamental to those systems and therefore more difficult to overcome.

It is important to consider a broad array of outcomes of societal concern when deciding how to reduce EID risk and not just those highlighted here. EIDs and mitigation interventions are likely to have profound consequences for people, the environment, wildlife and livestock, and these are very unlikely to provide uniform benefits. For example, within disease mitigation, it is plausible that prevention of emerging disease risks may compete, at least in resource terms, with endemic disease management. Beyond disease mitigation, the complete transition to high-yield systems (and corresponding loss of low-yield systems) could have profound consequences for social equity, if producers lacking the resources to invest in high-yield technologies are squeezed out of the market. Such an impact would be particularly damaging in societies where subsistence livestock production provides an economic safety net, vital nutrition or important cultural values [107]. These concerns, as well as EID risks, and broader considerations around environmental sustainability and food security will be heavily context dependent, and decisions over which interventions to pursue will need to be made at an appropriate level. One course of action to include concerns over EIDs into the broader discourse over sustainable food systems would be to integrate them into ecosystem service frameworks. This kind of framing would allow the mainstreaming of disease control into a holistic understanding of optimal farming practices.

There are concerns that countries where livestock production is expected to increase most steeply may be poorly placed to mitigate the risks of both higher and lower yielding approaches [7]. These limitations could constrain their capacity to prevent and respond rapidly to EIDs. However, there is considerable potential for improvements through investing in preventive practices, disease surveillance and regulation targeting particularly risk-prone activities such as multi-species housing or long-distance live transport. A key challenge for policy makers and researchers will be to identify logistically, politically and economically feasible interventions with the greatest potential to reduce risks in priority regions before diseases emerge. The costs of preventing a pandemic may be substantially less than the economic and human health costs of responding to one [41]. We anticipate that effective prevention will require resource transfers from high- to low- and middle-income countries.

## 9. Conclusion and evidence gaps

Our overview of the limited evidence available suggests that calls to reduce ‘intensive’ livestock farming practices to mitigate EID risk are premature and largely overlook two major issues. First, less ‘intensive’ systems are liable to be low-yielding. This means they require both a larger livestock population and more land and hence greater habitat loss and degradation, increasing the risk of zoonotic EID emergence. Second, there are considerable gaps in our understanding of many of the key factors that influence the relative EID risk of contrasting livestock systems. In our view, the most pressing unanswered questions include:

— What are the major entry and exit points for EIDs onto and off a farm? What biosecurity practices best mitigate these?
— How can surveillance be best adapted to livestock systems to maximize early detection of potential EIDs?
— How can we boost livestock health and welfare without compromising yields or biosecurity?
— What is the relationship between livestock genetic diversity and disease emergence?
— How does the density and pathogen burden of high-risk wild species vary across higher and lower yielding production landscapes?
— What extent, distribution and condition of natural habitats is needed to reduce EID risks to acceptable levels?
— Do the relative EID risks of higher versus lower yielding approaches to meeting demand change with overall production—and if so, is there a level of production where the least bad approach switches?
— Perhaps most importantly, how do risk factors interact? How far does improvement in one mitigate or enhance others?

These gaps should, we suggest, serve as a call to action. Contrary to some claims [16–20], we conclude that we currently lack adequate information to reach a robust view of the relationship between contrasting livestock systems and EID risk. Without further progress in filling the evidence gaps, we have identified here we argue it is impossible to make valid comparisons of alternatives matched by overall level of production and hence to draw defensible conclusions about which is likely to offer the best route to mitigating EID risk. We are in danger of making decisions that are ineffective and even counterproductive.

## Data Availability

The data are provided in the electronic supplementary material [108].

## References

[1] Jones KE, Patel NG, Levy MA, Storeygard A, Balk D, Gittleman JL, Daszak P. 2008 Global trends in emerging infectious diseases. Nature **451**, 990-993. (10.1038/nature06536) 18288193 PMC5960580

[2] Plowright RK, Parrish CR, McCallum H, Hudson PJ, Ko AI, Graham AL, Lloyd-Smith JO. 2017 Pathways to zoonotic spillover. Nat. Rev. Microbiol. **15**, 502-510. (10.1038/nrmicro.2017.45) 28555073 PMC5791534

[3] Johnson CK, Hitchens PL, Pandit PS, Rushmore J, Evans TS, Young CC, Doyle MM. 2020 Global shifts in mammalian population trends reveal key predictors of virus spillover risk. Proc. R. Soc. B **287**, 20192736. (10.1098/rspb.2019.2736) PMC7209068 32259475

[4] FAOSTAT. 2018 *Food and agriculture data* (1 November 2018). See https://www.fao.org/faostat/en/#home (accessed in 2021).

[5] Daszak P, Cunningham AA, Hyatt AD. 2001 Anthropogenic environmental change and the emergence of infectious diseases in wildlife. Acta Trop. **78**, 103-116. (10.1016/S0001-706X(00)00179-0) 11230820

[6] Macleod M, Gerber P, Mottet A, Tempio G, Falcucci A, Opio C, Vellinga T, Henderson B, Steinfeld H. 2013 Greenhouse gas emissions from pig and chicken supply chains – a global life cycle assessment. Rome, Italy: Food and Agricultural Organization of the United Nations (FAO).

[7] Gilbert W, Thomas L, Coyne L, Rushton J. 2020 Review: mitigating the risks posed by intensification in livestock production: the examples of antimicrobial resistance and zoonoses. Animal **15**, 100123. 33573940 10.1016/j.animal.2020.100123

[8] Pendrill F, Persson UM, Godar J, Kastner T. 2019 Deforestation displaced: trade in forest-risk commodities and the prospects for a global forest transition. Environ. Res. Lett. **14**, 055003. (10.1088/1748-9326/ab0d41)

[9] Faust CL, McCallum HI, Bloomfield LS, Gottdenker NL, Gillespie TR, Torney CJ, Dobson AP, Plowright RK. 2018 Pathogen spillover during land conversion. Ecol. Lett. **21**, 471-483. (10.1111/ele.12904) 29466832

[10] Espinosa R, Tago D, Treich N. 2020 Infectious diseases and meat production. Environ. Resour. Econ. **76**, 1-26. (10.1007/s10640-020-00484-3) PMC7399585 32836843

[11] González N, Marquès M, Nadal M, Domingo JL. 2020 Meat consumption: which are the current global risks? A review of recent (2010–2020) evidences. Food Res. Int. **137**, 109341. (10.1016/j.foodres.2020.109341) 33233049 PMC7256495

[12] Petrikova I, Cole J, Farlow A. 2020 COVID-19, wet markets, and planetary health. Lancet Planet. Heal. **4**, e213-e214. (10.1016/S2542-5196(20)30122-4) PMC7832206 32559435

[13] Herrero M, Thornton PK, Gerber P, Reid RS. 2009 Livestock, livelihoods and the environment: understanding the trade-offs. Curr. Opin. Environ. Sustain. **1**, 111-120. (10.1016/j.cosust.2009.10.003)

[14] Tilman D, Balzer C, Hill J, Befort BL. 2011 Global food demand and the sustainable intensification of agriculture. Proc. Natl Acad. Sci. USA **108**, 20 260-20 264. (10.1073/pnas.1116437108) PMC3250154 22106295

[15] Godfray HCJ et al. 2010 Food security: the challenge of feeding 9 billion people. Science **327**, 812-818. (10.1126/science.1185383) 20110467

[16] Pulliam JRC et al. 2012 Agricultural intensification, priming for persistence and the emergence of Nipah virus: a lethal bat-borne zoonosis. J. R. Soc. Interface **9**, 89-101. (10.1098/rsif.2011.0223) 21632614 PMC3223631

[17] Jones BA et al. 2013 Zoonosis emergence linked to agricultural intensification and environmental change. Proc. Natl Acad. Sci. USA **110**, 8399-8404. (10.1073/pnas.1208059110) 23671097 PMC3666729

[18] Liverani M et al. 2013 Understanding and managing zoonotic risk in the new livestock industries. Environ. Health Perspect. **121**, 873-877. (10.1289/ehp.1206001) 23665854 PMC3734490

[19] Hassell JM, Begon M, Ward MJ, Fèvre EM. 2017 Urbanization and disease emergence: dynamics at the wildlife–livestock–human interface. Trends Ecol. Evol. **32**, 55-67. (10.1016/j.tree.2016.09.012) 28029378 PMC5214842

[20] Mourkas E et al. 2020 Agricultural intensification and the evolution of host specialism in the enteric pathogen *Campylobacter jejuni*. Proc. Natl Acad. Sci. USA **117**, 11 018-11 028. (10.1073/pnas.1917168117) PMC7245135 32366649

[21] Borlaug N. 2007 Feeding a hungry world. Science **318**, 359. (10.1126/science.1151062) 17947551

[22] Di Marco M et al. 2020 Sustainable development must account for pandemic risk. Proc. Natl Acad. Sci. USA **117**, 3888-3892. (10.1073/pnas.2001655117) 32060123 PMC7049118

[23] Roos E, Bajželj B, Smith P, Patel M, Little D, Garnett T. 2017 Greedy or needy? Land use and climate impacts of food in 2050 under different livestock futures. Glob. Environ. Chang. **47**, 1-12. (10.1016/j.gloenvcha.2017.09.001)

[24] Bajželj B, Richards KS, Allwood JM, Smith P, Dennis JS, Curmi E, Gilligan CA. 2014 Importance of food-demand management for climate mitigation. Nat. Clim. Chang. **4**, 924-929. (10.1038/nclimate2353)

[25] Williams DR, Clark M, Buchanan GM, Ficetola GF, Rondinini C, Tilman D. 2020 Proactive conservation to prevent habitat losses to agricultural expansion. Nat. Sustain. **4**, 1-9. (10.1038/s41893-020-00656-5)

[26] Strassburg BBN, Latawiec AE, Barioni LG, Nobre CA, Da Silva VP, Valentim JF, Vianna M, Assad ED. 2014 When enough should be enough: improving the use of current agricultural lands could meet production demands and spare natural habitats in Brazil. Glob. Environ. Chang. **28**, 84-97. (10.1016/j.gloenvcha.2014.06.001)

[27] zu Ermgassen EKHJ et al. 2018 Results from on-the-ground efforts to promote sustainable cattle ranching in the Brazilian Amazon. Sustainability **10**, 1301. (10.3390/su10041301)

[28] Smolinski MS, Hamburg MA, Lederberg J. 2003 Microbial threats to health: emergence, detection, and response, Washington, DC: The National Academies Press. 25057653

[29] Petrovan SO et al. 2021 Post COVID-19: a solution scan of options for preventing future zoonotic epidemics. Biol. Rev. Camb. Philos. Soc. **96**, 12 774. 10.1111/brv.12774 PMC8444924 34231315

[30] Rohr JR et al. 2019 Emerging human infectious diseases and the links to global food production. Nat. Sustain. **2**, 445-456. (10.1038/s41893-019-0293-3) 32219187 PMC7091874

[31] Booth H et al. 2021 Investigating the risks of removing wild meat from global food systems. Curr. Biol. **31**, 1788-1797. (10.1016/j.cub.2021.01.079) 33607034 PMC8094154

[32] Balmford A et al. 2018 The environmental costs and benefits of high-yield farming. Nat. Sustain. **1**, 477-485. (10.1038/s41893-018-0138-5) 30450426 PMC6237269

[33] Phalan B, Onial M, Balmford A, Green RE. 2011 Reconciling food production and biodiversity conservation: land sharing and land sparing compared. Science **333**, 1289-1291. (10.1126/science.1208742) 21885781

[34] Hulme MF et al. 2013 Conserving the birds of Uganda's banana-coffee arc: land sparing and land sharing compared. PLoS ONE **8**, 54597. (10.1371/journal.pone.0054597) PMC3563584 23390501

[35] Balmford A, Green R, Phalan B. 2015 Land for food & land for nature? Daedalus **144**, 57-75. (10.1162/DAED_a_00354)

[36] Kamp J, Urazaliev R, Balmford A, Donald PF, Green RE, Lamb AJ, Phalan B. 2015 Agricultural development and the conservation of avian biodiversity on the Eurasian steppes: a comparison of land-sparing and land-sharing approaches. J. Appl. Ecol. **52**, 1578-1587. (10.1111/1365-2664.12527)

[37] Dotta G, Phalan B, Silva TW, Green R, Balmford A. 2016 Assessing strategies to reconcile agriculture and bird conservation in the temperate grasslands of South America. Conserv. Biol. **30**, 618-627. (10.1111/cobi.12635) 26400720

[38] Williams DR, Alvarado F, Green RE, Manica A, Phalan B, Balmford A. 2017 Land-use strategies to balance livestock production, biodiversity conservation and carbon storage in Yucatán, Mexico. Glob. Chang. Biol. **23**, 5260-5272. (10.1111/gcb.13791) 28614629

[39] Balmford A. 2021 Concentrating vs. spreading our footprint: how to meet humanity's needs at least cost to nature. J. Zool. **315**, 79-109. (10.1111/jzo.12920)

[40] Pike J, Bogich T, Elwood S, Finnoff DC, Daszak P. 2014 Economic optimization of a global strategy to address the pandemic threat. Proc. Natl Acad. Sci. USA **111**, 18 519-18 523. (10.1073/pnas.1412661112) PMC4284561 25512538

[41] Dobson AP et al. 2020 Ecology and economics for pandemic prevention. Science **369**, 379-381. (10.1126/science.abc3189) 32703868

[42] Judge J, McDonald RA, Walker N, Delahay RJ. 2011 Effectiveness of biosecurity measures in preventing badger visits to farm buildings. PLoS ONE **6**, e28941. (10.1371/journal.pone.0028941) 22220199 PMC3248415

[43] Rule AM, Evans SL, Silbergeld EK. 2008 Food animal transport: a potential source of community exposures to health hazards from industrial farming (CAFOs). J. Infect. Public Health **1**, 33-39. (10.1016/j.jiph.2008.08.001) 20701843

[44] Nelson MI et al. 2015 Global migration of influenza A viruses in swine. Nat. Commun. **6**, 1-11. (10.1038/ncomms7696) PMC4380236 25813399

[45] Brennan ML, Christley RM. 2012 Biosecurity on cattle farms: a study in north-west England. PLoS ONE **7**, e28139. (10.1371/journal.pone.0028139) 22235244 PMC3250388

[46] Van Steenwinkel S, Ribbens S, Ducheyne E, Goossens E, Dewulf J. 2011 Assessing biosecurity practices, movements and densities of poultry sites across Belgium, resulting in different farm risk-groups for infectious disease introduction and spread. Prev. Vet. Med. **98**, 259-270. (10.1016/j.prevetmed.2010.12.004) 21195492

[47] Hamilton-West C, Rojas H, Pinto J, Orozco J, Hervé-Claude LP, Urcelay S. 2012 Characterization of backyard poultry production systems and disease risk in the central zone of Chile. Res. Vet. Sci. **93**, 121-124. (10.1016/j.rvsc.2011.06.015) 21752410

[48] Temple D, Manteca X. 2020 Animal welfare in extensive production systems is still an area of concern. Front. Sustain. Food Syst. **4**, 545902. (10.3389/fsufs.2020.545902)

[49] Otte J, Pfeiffer D, Tiensin T, Price L, Silbergeld E. 2007 Highly pathogenic avian influenza risk, biosecurity and smallholder adversity. Livest. Res. Rural Dev. **19**, 102.

[50] Delpont M, Guinat C, Guérin JL, Vaillancourt JP, Paul MC. 2021 Biosecurity measures in French poultry farms are associated with farm type and location. Prev. Vet. Med. **195**, 105466. (10.1016/j.prevetmed.2021.105466) 34419776

[51] Robertson ID, Control D. 2020 Prevention and on-farm biosecurity: the role of veterinary epidemiology. Engineering **6**, 20-25. (10.1016/j.eng.2019.10.004)

[52] Karabozhilova I, Wieland B, Alonso S, Salonen L, Häsler B. 2012 Backyard chicken keeping in the Greater London urban area: welfare status, biosecurity and disease control issues. Br. Poult. Sci. **53**, 421-430. (10.1080/00071668.2012.707309) 23130576

[53] Tomley FM, Shirley MW. 2009 Livestock infectious diseases and zoonoses. Phil. Trans. R. Soc. B **364**, 2637-2642. (10.1098/rstb.2009.0133) 19687034 PMC2865087

[54] Chan JFW, To KKW, Tse H, Jin DY, Yuen KY. 2013 Interspecies transmission and emergence of novel viruses: lessons from bats and birds. Trends Microbiol. **21**, 544-555. (10.1016/j.tim.2013.05.005) 23770275 PMC7126491

[55] Van Wagenberg CPA, De Haas Y, Hogeveen H, Van Krimpen MM, Meuwissen MP, Van Middelaar CE, Rodenburg TB. 2017 Animal board invited review: comparing conventional and organic livestock production systems on different aspects of sustainability. Animal **11**, 1839-1851. (10.1017/S175173111700115X) 28558861 PMC5607874

[56] Graham JP, Leibler JH, Price LB, Otte JM, Pfeiffer DU, Tiensin T, Silbergeld EK. 2008 The animal-human interface and infectious disease in industrial food animal production: rethinking biosecurity and biocontainment. Public Health Rep. **123**, 282-299. (10.1177/003335490812300309) 19006971 PMC2289982

[57] Dhingra MS et al. 2018 Geographical and historical patterns in the emergences of novel highly pathogenic avian influenza (HPAI) H5 and H7 viruses in poultry. Front. Vet. Sci. **5**, 84. (10.3389/fvets.2018.00084) 29922681 PMC5996087

[58] Heft-Neal S et al. 2008 *Pro-poor livestock policy initative*. A living from livestock report. Supply chain auditing for poultry production in Thailand. Rome, Italy: Food and Agricultural Organization of the United Nations (FAO).

[59] Robinson TP et al. 2014 Mapping the global distribution of livestock. PLoS ONE **9**, e96084. (10.1371/journal.pone.0096084) 24875496 PMC4038494

[60] Rajão R et al. 2020 The rotten apples of Brazil's agribusiness. Science **369**, 246-248. (10.1126/science.aba6646) 32675358

[61] Conlan AJK, McKinley TJ, Karolemeas K, Pollock EB, Goodchild AV, Mitchell AP, Birch CP, Clifton-Hadley RS, Wood JL. 2012 Estimating the hidden burden of bovine tuberculosis in Great Britain. PLoS Comput. Biol. **8**, e1002730. (10.1371/journal.pcbi.1002730) 23093923 PMC3475695

[62] Pitzer VE, Aguas R, Riley S, Loeffen WL, Wood JL, Grenfell BT. 2016 High turnover drives prolonged persistence of influenza in managed pig herds. J. R. Soc. Interface **13**, 20160138. (10.1098/rsif.2016.0138) 27358277 PMC4938081

[63] Fuller TL et al. 2013 Predicting hotspots for influenza virus reassortment. Emerg. Infect. Dis. **19**, 581-588. (10.3201/eid1904.120903) 23628436 PMC3647410

[64] Maes D, Deluyker H, Verdonck M, Castryck F, Miry C, Vrijens B, De Kruif A. 2000 Herd factors associated with the seroprevalences of four major respiratory pathogens in slaughter pigs from farrow-to-finish pig herds. Vet. Res. **31**, 313-327. (10.1051/vetres:2000122) 10863948

[65] Cressler CE, McLeod DV, Rozins C, Van Den Hoogen J, Day T. 2016 The adaptive evolution of virulence: a review of theoretical predictions and empirical tests. Parasitology **143**, 915-930. (10.1017/S003118201500092X) 26302775 PMC4873896

[66] Lenski RE, May RM. 1994 The evolution of virulence in parasites and pathogens: reconciliation between two competing hypotheses. J. Theor. Biol. **169**, 253-265. (10.1006/jtbi.1994.1146) 7967617

[67] Rozins C, Day T. 2017 The industrialization of farming may be driving virulence evolution. Evol. Appl. **10**, 189. (10.1111/eva.12442) 28127395 PMC5253429

[68] Rostagno MH. 2009 Can stress in farm animals increase food safety risk? Foodborne Pathog. Dis. **6**, 767-776. (10.1089/fpd.2009.0315) 19737056

[69] De Passillé AM, Rushen J. 2005 Food safety and environmental issues in animal welfare. OIE Rev. Sci. Tech. **24**, 757-766. (10.20506/rst.24.2.1599) 16358525

[70] Hayek MN, Garrett RD. 2018 Nationwide shift to grass-fed beef requires larger cattle population. Environ. Res. Lett. **13**, 84005. (10.1088/1748-9326/aad401)

[71] Meadows AJ, Mundt CC, Keeling MJ, Tildesley MJ. 2018 Disentangling the influence of livestock vs. farm density on livestock disease epidemics. Ecosphere **9**, e02294. (10.1002/ecs2.2294)

[72] CIWF 2021. *Animal cruelty | Compassion in World Farming* (2 July 2021). See https://www.ciwf.org.uk/factory-farming/animal-cruelty/ (accessed in 2021).

[73] FAWC. 2018 Evidence and the welfare of farmed animals part 2: evidence-based decision making. London, UK: Farm Animal Welfare Committee. See https://assets.publishing.service.gov.uk/government/uploads/system/uploads/attachment_data/file/727191/fawc-evidence-part2-farmed-animals.pdf.

[74] Dawkins MS. 2012 Commercial scale research and assessment of poultry welfare. Br. Poult. Sci. **53**, 1-6. (10.1080/00071668.2011.628640) 22404799

[75] Guy JH, Rowlinson P, Chadwick JP, Ellis M. 2002 Health conditions of two genotypes of growing-finishing pig in three different housing systems: implications for welfare. Livest. Prod. Sci. **75**, 233-243. (10.1016/S0301-6226(01)00327-X)

[76] Fu L et al. 2016 Stocking density affects welfare indicators of growing pigs of different group sizes after regrouping. Appl. Anim. Behav. Sci. **174**, 42-50. (10.1016/j.applanim.2015.10.002)

[77] Nicol CJ, Gregory NG, Knowles TG, Parkman ID, Wilkins LJ. 1999 Differential effects of increased stocking density, mediated by increased flock size, on feather pecking and aggression in laying hens. Appl. Anim. Behav. Sci. **65**, 137-152. (10.1016/S0168-1591(99)00057-X)

[78] Buijs S, Keeling L, Rettenbacher S, van Poucke E, Tuyttens FAM. 2009 Stocking density effects on broiler welfare: identifying sensitive ranges for different indicators. Poult. Sci. **88**, 1536-1543. (10.3382/ps.2009-00007) 19590066

[79] Costa JHC, Hötzel MJ, Longo C, Balcão LF. 2013 A survey of management practices that influence production and welfare of dairy cattle on family farms in southern Brazil. J. Dairy Sci. **96**, 307-317. (10.3168/jds.2012-5906) 23102960

[80] Dawkins MS. 2017 Animal welfare and efficient farming: is conflict inevitable? Anim. Prod. Sci. **57**, 201. (10.1071/AN15383)

[81] Schneider DS, Ayres JS. 2008 Two ways to survive infection: what resistance and tolerance can teach us about treating infectious diseases. Nat. Rev. Immunol. **8**, 889-895. (10.1038/nri2432) 18927577 PMC4368196

[82] FAO 2007 *The state of the world's animal genetic resources for food and agriculture.* Rome, Italy: FAO.

[83] Vordermeier M, Ameni G, Berg S, Bishop R, Robertson BD, Aseffa A, Hewinson RG, Young DB. 2012 The influence of cattle breed on susceptibility to bovine tuberculosis in Ethiopia. Comp. Immunol. Microbiol. Infect. Dis. **35**, 227-232. (10.1016/j.cimid.2012.01.003) 22304898 PMC3339321

[84] Pal A, Pal A, Mallick AI, Biswas P, Chatterjee PN. 2020 Molecular characterization of Bu-1 and TLR2 gene in Haringhata Black chicken. Genomics **112**, 472-483. (10.1016/j.ygeno.2019.03.010) 30902756

[85] Wang H et al. 2016 High genetic diversity and frequent genetic reassortment of avian influenza A(H9N2) viruses along the East Asian-Australian migratory flyway. Infect. Genet. Evol. **39**, 325-329. (10.1016/j.meegid.2016.02.013) 26876220

[86] UNEP 2016 *Zoonoses: blurred lines of emergent disease and ecosystem health*. UNEP. See https://wedocs.unep.org/handle/20.500.11822/32060.

[87] Everard M, Johnston P, Santillo D, Staddon C. 2020 The role of ecosystems in mitigation and management of Covid-19 and other zoonoses. Environ. Sci. Policy **111**, 7-17. (10.1016/j.envsci.2020.05.017) 32501392 PMC7247996

[88] Notter DR. 1999 The importance of genetic diversity in livestock populations of the future. J. Anim. Sci. **77**, 61-69. (10.2527/1999.77161x) 10064028

[89] Groeneveld LF et al. 2010 Genetic diversity in farm animals – a review. Anim. Genet. **41**, 6-31. (10.1111/j.1365-2052.2010.02038.x) 20500753

[90] Thornton PK. 2010 Livestock production: recent trends, future prospects. Phil. Trans. R. Soc. B **365**, 2853-2867. (10.1098/rstb.2010.0134) 20713389 PMC2935116

[91] Mpenda FN, Schilling MA, Campbell Z, Mngumi EB, Buza J. 2019 The genetic diversity of local African chickens: a potential for selection of chickens resistant to viral infections. J. Appl. Poult. Res. **28**, 1-12. (10.3382/japr/pfy063)

[92] Pal A, Chakravarty AK. 2020 Disease resistance for different livestock species. In Genetics and breeding for disease resistance of livestock. Amsterdam, The Netherlands: Elsevier, pp. 271-296.

[93] Becker DJ, Albery GF, Kessler MK, Lunn TJ, Falvo CA, Czirják GÁ, Martin LB, Plowright RK. 2020 Macroimmunology: the drivers and consequences of spatial patterns in wildlife immune defence. J. Anim. Ecol. **89**, 972-995. (10.1111/1365-2656.13166) 31856309 PMC7138727

[94] Gibb R, Redding DW, Chin KQ, Donnelly CA, Blackburn TM, Newbold T, Jones KE. 2020 Zoonotic host diversity increases in human-dominated ecosystems. Nature **584**, 398-402. (10.1038/s41586-020-2562-8) 32759999

[95] Plowright RK, Reaser J, Locke H, Woodley SJ, Patz JA, Becker D, Oppler G, Hudson P, Tabor GM. 2021 Land use-induced spillover: a call to action to safeguard environmental, animal, and human health. The Lancet Planetary Health **5**, 237-245. (10.1016/S2542-5196(21)00031-0) PMC7935684 33684341

[96] Páez DJ, Giles J, McCallum H, Field H, Jordan D, Peel AJ, Plowright RK. 2017 Conditions affecting the timing and magnitude of Hendra virus shedding across pteropodid bat populations in Australia. Epidemiol. Infect. **145**, 3143-3153. (10.1017/S0950268817002138) 28942750 PMC5783192

[97] Despommier D, Ellis BR, Wilcox BA. 2006 The role of ecotones in emerging infectious diseases. Ecohealth **3**, 281-289. (10.1007/s10393-006-0063-3)

[98] Rulli MC, D'Odorico P, Galli N, Hayman D. 2021 Land use change and the livestock revolution increase the risk of zoonotic coronavirus transmission from rhinolopid bats Nature Food **2**, 409-416. (10.1038/s43016-021-00285-x) 37118224

[99] Prist PR, Prado A, Tambosi LR, Umetsu F, de Arruda Bueno A, Pardini R, Metzger JP. 2021 Moving to healthier landscapes: forest restoration decreases the abundance of Hantavirus reservoir rodents in tropical forests. Sci. Total Environ. **752**, 141967. (10.1016/j.scitotenv.2020.141967) 32892056

[100] Morand S, Lajaunie C. 2021 Outbreaks of vector-borne and zoonotic diseases are associated with changes in forest cover and oil palm expansion at global scale. Front. Vet. Sci. **8**, 661063. (10.3389/fvets.2021.661063) 33842581 PMC8024476

[101] Nicoletti M, Murugan K, Benelli G. 2016 Emerging insect-borne diseases of agricultural, medical and veterinary importance. In Insecticides resistance, pp. 219-243. USA: InTech. (10.5772/61467)

[102] Lovera R, Fernández MS, Cavia R. 2019 Small rodent species on pig and dairy farms: habitat selection and distribution. Pest Manag. Sci. **75**, 1234-1241. (10.1002/ps.5299) 30536608

[103] Chua KB. 2003 Nipah virus outbreak in Malaysia. J. Clin. Virol. **26**, 265-275. (10.1016/S1386-6532(02)00268-8) 12637075

[104] McKee CD, Islam A, Luby SP, Salje H, Hudson PJ, Plowright RK, Gurley ES. 2021 The ecology of Nipah virus in Bangladesh: a nexus of land-use change and opportunistic feeding behavior in bats. Viruses **13**, 169. (10.3390/v13020169) 33498685 PMC7910977

[105] Kessler MK et al. 2018 Changing resource landscapes and spillover of henipaviruses. Ann. N. Y. Acad. Sci. **1429**, 78-99. (10.1111/nyas.13910) 30138535 PMC6778453

[106] Phalan B et al. 2016 How can higher-yield farming help to spare nature? Science **351**, 450-451. (10.1126/science.aad0055) 26823413

[107] Herrero M, Grace D, Njuki J, Johnson N, Enahoro D, Silvestri S, Rufino MC. 2013 The roles of livestock in developing countries. Animal **7**, 3-18. (10.1017/S1751731112001954) 23121696

[108] Bartlett H, Holmes MA, Petrovan SO, Williams DR, Wood JLN, Balmford A. 2022 Understanding the relative risks of zoonosis emergence under contrasting approaches to meeting livestock product demand. FigShare. (10.6084/m9.figshare.c.6049391) PMC9214290 35754996

